# Genome-Wide Mapping of Quantitative Trait Loci Conferring All-Stage and High-Temperature Adult-Plant Resistance to Stripe Rust in Spring Wheat Landrace PI 181410

**DOI:** 10.3390/ijms21020478

**Published:** 2020-01-12

**Authors:** Yan Liu, Yanmin Qie, Xing Li, Meinan Wang, Xianming Chen

**Affiliations:** 1 Department of Plant Pathology, Washington State University, Pullman, WA 99164-6430, USA; y.liu@wsu.edu (Y.L.); qieyanmin2014@163.com (Y.Q.); lixing@hebau.edu.cn (X.L.); meinan_wang@wsu.edu (M.W.); 2 Institute of Cereal and Oil Crops, Hebei Academy of Agricultural and Forestry Sciences, 162 Hengshan Street, Gaoxin District, Shijiazhuang, Hebei 050035, China; 3 College of Plant Protection, Hebei Agricultural University, Baoding, Hebei 071001, China; 4 US Department of Agriculture, Agricultural Research Service, Wheat Health, Genetics, and Quality Research Unit, Pullman, WA 99164-6430, USA

**Keywords:** disease resistance, *Puccinia striiformis*, QTL mapping, stripe rust, *Triticum aestivum*, wheat

## Abstract

Stripe rust, caused by *Puccinia striiformis* f. sp. *tritici* (*Pst*), is one of the most destructive diseases of wheat in the world. Genetic resistance is the best strategy for control of the disease. Spring wheat landrace PI 181410 has shown high level resistance to stripe rust. The present study characterized the landrace to have both race-specific all-stage resistance and nonrace-specific high-temperature adult-plant (HTAP) resistance. To map quantitative trait loci (QTL) for the resistance in PI 181410, it was crossed with Avocet S (AvS), from which a recombinant inbred line population was developed. The F_5_–F_8_ populations were consecutively phenotyped for stripe rust response in multiple field environments under natural *Pst* infection, and the F_7_ population was phenotyped in seedlings at low temperature and in adult-plant stage with selected *Pst* races in the greenhouse. The F_7_ population was genotyped using the 90K wheat SNP chip. Three QTL, *QYrPI181410.wgp-4AS*, *QYrPI181410.wgp-4BL*, and *QYrPI181410.wgp-5BL*.1, from PI 181410 for all-stage resistance, were mapped on chromosome arms 4AS, 4BL, and 5BL, respectively. Four QTL, *QYrPI181410.wgp-1BL*, *QYrPI181410.wgp-4BL*, *QYrPI181410.wgp-5AS*, and *QYrPI181410.wgp-5BL.2*, were identified from PI 181410 for HTAP resistance and mapped to 1BL, 4BL, 5AS, and 5BL, respectively. Two QTL with minor effects on stripe rust response were identified from AvS and mapped to 2BS and 2BL. Four of the QTL from PI 181410 and one from AvS were potentially new. As the 4BL QTL was most effective and likely a new gene for stripe rust resistance, three kompetitive allele specific PCR (KASP) markers were developed for incorporating this gene into new wheat cultivars.

## 1. Introduction

Wheat (*Triticum aestivum* L.) is one of the most important food crops worldwide, as it provides humans most of their daily calories and proteins. With the growing of the human population, wheat demand is expected to increase by 66% in 2040 [1]. However, wheat production has been continually threatened by abiotic and biotic stresses. Stripe rust (also known as yellow rust), caused by fungal pathogen *Puccinia striiformis* Westend. f. sp. *tritici* Erikss. (*Pst*), is especially a devastating disease in the world [2,3], and the fungus is ranked among the global burden pathogens and pests of major crops [4]. The disease can cause more than 90% yield losses in fields grown with susceptible cultivars if the weather conditions are extremely favorable to stripe rust development [5].

Growing resistant cultivars and application of fungicides are two major approaches for control of stripe rust. Use of fungicides adds significant cost to production; has potential to select fungicide-tolerant populations of the pathogen; is potentially harmful to humans, animals, and the environment; and therefore, is limited in organic farming and areas under protection for wildlife habitats. In contrast, growing resistant cultivars does not have these problems, but is easy to use, efficient, and more effective for control of stripe rust [6,7]. On the other hand, resistance may have some other problems. For example, resistant cultivars may become susceptible to new virulent races of the pathogen or the resistance level may not be adequate when the disease is severe. However, these problems can be solved by developing cultivars with durable, high-level resistance [5].

Resistance to stripe rust is generally described in two types: all-stage resistance and high-temperature adult-plant (HTAP) resistance [2,7]. All-stage resistance (ASR), also called seedling resistance, is normally controlled by a single gene that provides high-level resistance throughout the growth stages. However, ASR is usually race-specific, and cultivars with this type of resistance can be easily overcome by new virulent races of the pathogen. More than 300 *Pst* races have been identified from wheat stripe rust samples collected from the US and several countries, and the recent predominant races in the US are virulent on 16 of the 18 *Yr* single-gene lines in the Avocet background that are used to differentiate *Pst* races [8,9,10]. In contrast, HTAP resistance, which is expressed when the plant grows old and weather becomes warm, is nonrace-specific and durable [7,11]. The quantitative nature makes HTAP resistance breeding relatively difficult compared to ASR, and also often the partial resistance may not provide adequate control if the disease starts in the early growth stage and the weather conditions favor the disease but do not favor expression of HTAP resistance. Therefore, it is better to combine both types of resistance by pyramiding genes for effective ASR and HTAP resistance to utilize their advantages and overcome the disadvantages. This approach requires a large number of genes with user-friendly markers for marker-assisted selection (MAS). Thus, mapping new resistance genes is a continual task for developing wheat cultivars with durable, high-level resistance to stripe rust to achieve sustainable and more effective control.

Wheat landraces are valuable sources of resistance to stripe rust. PI 181410, a spring wheat landrace originally from Afghanistan (https://npgsweb.ars-grin.gov/gringlobal/accessiondetail.aspx?id=1157002), has shown excellent resistance to stripe rust [12]. However, the genes for resistance to stripe rust in PI 181410 were not known. Therefore, the objectives of the present study were to (1) characterize stripe rust resistance in PI 181410, (2) identify and map genes or quantitative trait loci (QTL) conferring resistance, and (3) develop molecular markers for marker-assisted selection.

## 2. Results

### 2.1. Characterization of Stripe Rust Resistance in PI 181410

In the field, PI 181410 has always shown resistance with infection type (IT) 1–2 and severity (SE) 5%–20% to stripe rust at adult-plant stage in both Mount Vernon and Pullman, Washington, USA for 13 years since 2006 (Figure S1). In contrast, the susceptible check, AvS, has been always highly susceptible (IT 8, SE 90%–100%) in the same nurseries. In the greenhouse tests, the seedlings of PI 181410 were resistant (IT 3) to *Pst* race PSTv-4, moderately resistant (IT 5) to PSTv-14, and susceptible (IT 8) to races PSTv-37, PSTv-40, and PSTv-51 in the low-temperature (LT) (4–20 °C) tests (Table 1). In the high-temperature (HT) (10–30 °C) tests, PI 181410 seedlings were highly resistant (IT 2) to PSTv-4, PSTv-14, PSTv-40, and PSTv-51 but susceptible (IT 7) to PSTv-37. The adult-plants of PI 181410 showed high resistance (IT 2) to all the five tested races PSTv-4, PSTv-14, PSTv-37, PSTv-40, and PSTv-51 at both LT and HT. For comparison, the seedlings and adult plants of AvS were always highly susceptible (IT 8–9, SE 80%–100%) to all tested races at both LT and HT. The results indicated that PI 181410 has both race-specific ASR and nonrace-specific HTAP resistance.

### 2.2. Stripe Rust Phenotypes of the Recombinant Inbred Line (RIL) Population

**Figure 1:**
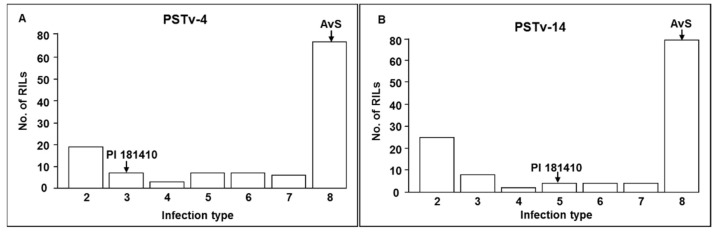
The distribution of infection types (ITs) among F_7_ recombinant inbred lines (RILs) derived from the cross AvS × PI 181410 tested in greenhouse using races (**A**) PSTv-4 and (**B**) PSTv-14 of *Puccinia striiformis* f. sp. *tritici*.

**Figure 2:**
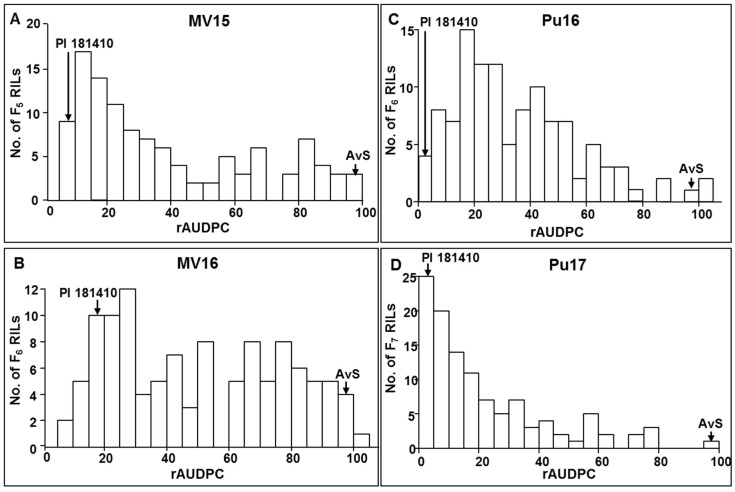
The distribution of relative area under the disease progress curve (rAUDPC) among recombinant inbred lines (RILs) derived from the cross AvS × PI 181410 in the field tests in Pullman and Mount Vernon, WA in 2015, 2016, and/or 2017.

The distributions of seedling IT among the 170 F_7_ RILs tested with both PSTv-4 and PSTv-14 were continuous, and the patterns were similar (Figure 1, Table S1). The F_5_, F_6_, and F_7_ RILs were tested under natural infections of *Pst* in the fields at Mount Vernon and Pullman in 2015, 2016, and 2017, respectively. The IT distributions of adult plants among the 170 RILs in field environments were also continuous but the patterns are different (Figure S2). Similarly, the relative area under the disease progress curve (rAUDPC) data also had continuous distributions with slightly different patterns (Figure 2). These results indicated that the stripe rust resistance in PI 181410 was quantitatively inherited. However, the distribution patterns were similar between the races tested in the greenhouse and were different among the year–location environments.

The analysis of variance (ANOVA) showed that the genetic and environmental effects and their interaction were all significant (*p* < 0.001) (Table 2). Broad-sense heritability (H^2^) was estimated as 0.68 for the rAUDPC data and 0.78 for IT data, indicating that the resistance in PI 181410 was highly heritable. The correlation coefficients of either IT or rAUDPC for all possible pairwise environment comparisons were highly significant (*p* < 0.001), but the values ranged from 0.29 to 0.79 (Table 3). Correlation coefficients were much higher between any two greenhouse tests or field tests than those between a field test and a greenhouse test. The phenotypical analyses indicated that different QTL controlled the resistances observed in different environments and suggested that QTL analysis should be used to map the genes controlling the resistance in the RIL population.

### 2.3. Genetic Linkage Groups

A total of 13,396 polymorphic single nucleotide polymorphism (SNP) markers were detected between parental lines AvS and PI 181410 using the 90K SNP chip. After removing SNPs that did not fit the 1:1 segregation ratio and duplicates of cosegregating SNPs, the final 3077 SNPs were used to construct linkage groups, and 20 linkage groups were obtained. The 20 linkage groups represent chromosomes 1A, 1B, 1D, 2A, 2B, 2D, 3A, 3B, 3D, 4A, 4B, 5A, 5B, 5D, 6A, 6B, 6D, 7A, 7B, and 7D (Table S2). The genetic map consisting of the 20 linkages is shown in Figure S3. The total genetic length was 2654 cM, and average genetic distance between two SNPs was 0.86 cM.

### 2.4. QTL for ASR to Stripe Rust

**Figure 3:**
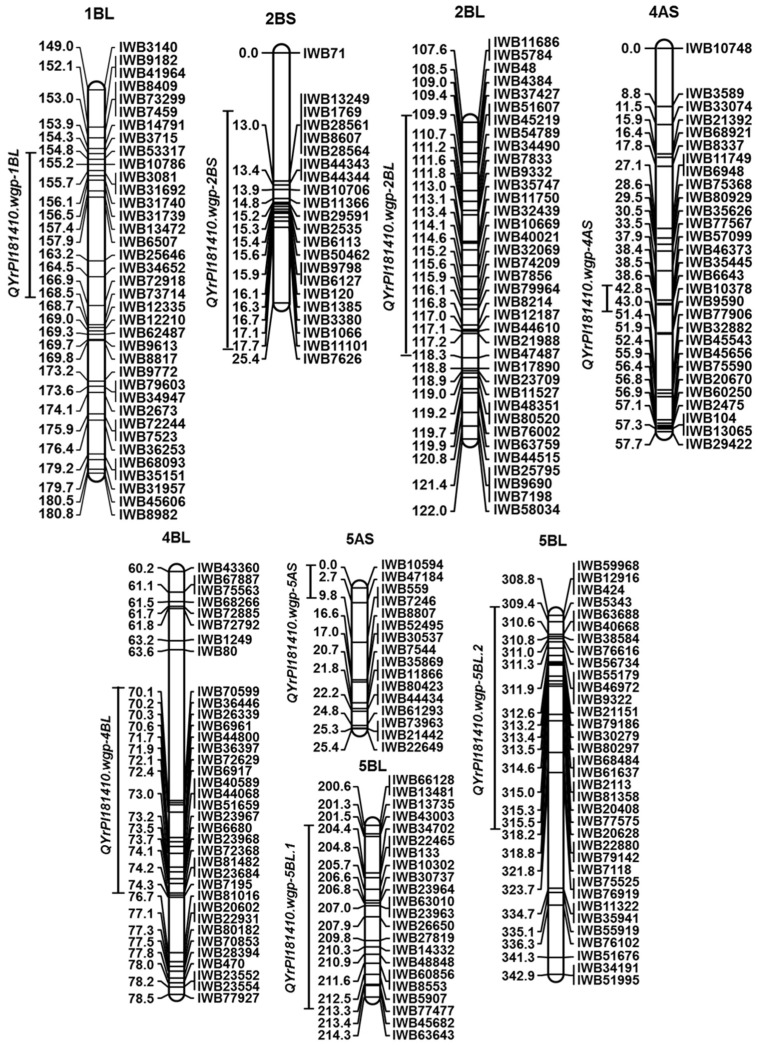
Genetic maps for the eight stripe rust resistance QTL identified in PI 181410 and AvS in this study. The solid bars on the left of each genetic map indicate the QTL position and interval.

Using the two sets of IT data of F_7_ RILs at the seedling stage tested with races PSTv-4 and PSTv-14 in the greenhouse, we detected three QTL, *QYrPI181410.wgp-4AS*, *QYrPI181410.wgp-4BL,* and *QYrPI181410.wgp-5BL.1*, conferring resistance to both races (Table 4, Figure 3).

*QYrPI181410.wgp-4AS* was detected by SNP *IWB9590* with the logarithm of odds (LOD) of 3.3 and 2.7 for PSTv-4 and PSTv-14, respectively (Table 4). It peaked at the genetic location of 43.0 cM and spanned a relatively small interval of 4.0 cM on the short arm of chromosome 4A. This QTL explained 11.2% and 5.4% of the phenotypic variation to PSTv-4 and PSTv-14, respectively.

*QYrPI181410.wgp-4BL* was detected by *IWB6961* on 4BL using two sets of IT data with both PSTv-4 and PSTv-14 with the LOD ranging from 2.5 to 3.6 (Table 4). It peaked at the location of 71.0 cM and spanned an interval of 20.0 cM region. This QTL explained 8.6% and 11.0% of the phenotypic variation to PSTv-4 and PSTv-14, respectively. *QYrPI181410.wgp-5BL.1* was detected by *IWB865* and peaked at the location of 211.0 cM with an interval of 10.0 cM on 5BL. It explained 15.1% and 14.0% of the phenotypic variation to PSTv-4 and PSTv-14, respectively.

### 2.5. QTL for HTAP Resistance

Using 12 sets of phenotypic data from field tests and two sets of adult-plant phenotypic data from the greenhouse tests (Table S1), we identified a total of six resistance QTL including *QYrPI181410.wgp-1BL*, *QYrAvS.wgp-2BS*, *QYrAvS.wgp-2BL*, *QYrPI181410.wgp-4BL*, *QYrPI181410.wgp-5AS*, and *QYrPI181410.wgp-5BL.2* (Table 5, Figure 3).

*QYrPI181410.wgp-1BL* was detected by *IWB6507* using four sets of field IT data at Mount Vernon in 2015, 2016, and 2017 and at Pullman in 2016, and one set of rAUDPC data from Mount Vernon in 2015. It explained 6.6% to 10.9% of the phenotypic variation (Table 5). The overlap interval of *QYrPI181410.wgp-1BL* spanned about 6.0 cM genomic region on 1BL with LOD ranging from 2.9 to 6.4 (Table 5).

**Figure 4:**
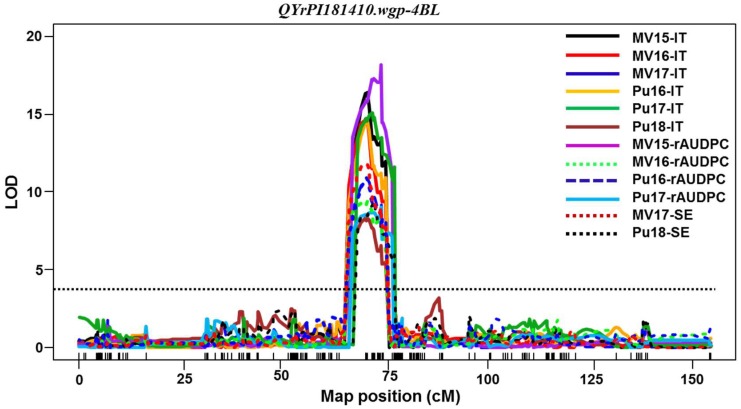
The LOD curves of *QYrPI181410.wgp-4BL* detected on chromosome 4B using 12 sets of data collected from the field tests across all environments including six sets of infection type (IT) data, four sets of relative area under the disease progress curve (rAUDPC) data, and two sets of disease severity (SE) data. The horizontal dotted line indicates the LOD threshold at 3.0.

A major QTL, *QYrPI181410.wgp-4BL*, was consistently detected using all 12 sets of phenotypic data from field tests and two sets of data from greenhouse tests. The LOD value of *QYrPI181410.wgp-4BL* ranged from 8.5 to 17.4 (Table 5, Figure 4). It peaked around the genetic position of 70.1 cM by SNP markers *IWB70599* and *IWB6961* within an interval of 3.0 cM (68.0 cM to 71.0 cM) on 4BL and explained 29.2% to 50.0% of the phenotypic variation. However, the phenotypic effect of *QYrPI181410.wgp-4BL* was lower (29.2 to 32.5) for infection type than for severity in Pullman, WA.

Two minor QTL, *QYrAvS.wgp-2BS* and *QYrAvS.wgp-2BL*, were detected on chromosome 2B and the effects were provided by the susceptible parent AvS (Table 5, Figure 3). *QYrAvS.wgp-2BS* was detected with two sets of IT data from Mount Vernon and explained 5.0%~5.6% of IT variation. *QYrAvS.wgp-2BL* was only detected with one set of IT data from the Pullman location and explained 7.2% of IT variation. Both QTL did not have any significant effect on rAUDPC.

*QYrPI181410.wgp-5AS* was identified with two sets of SE data as a minor QTL conferring resistance on reducing severity of stripe rust in PI 181410 (Table 5). This QTL was detected by *IWB47184* and located on the telomeric end of 5AS. It explained 3.9% to 4.2% of SE variation.

*QYrPI181410.wgp-5BL.2* was detected with five sets of field IT data from Mount Vernon in 2015 and 2016 and Pullman in 2016, 2017, and 2018 (Table 5, Figure 3). It was located within a 7.0 cM genomic interval and peaked around 310.0 cM close to the end of 5BL. This QTL explained 4.8% and 7.9% of the IT variation of data from Mount Vernon. However, it explained 14.6% to 17.3% of IT variation of data from Pullman, WA. This result suggested that the effect of *QYrPI181410.wgp-5BL.2* varies among the environments.

### 2.6. Effects of QTL Combinations

**Figure 5:**
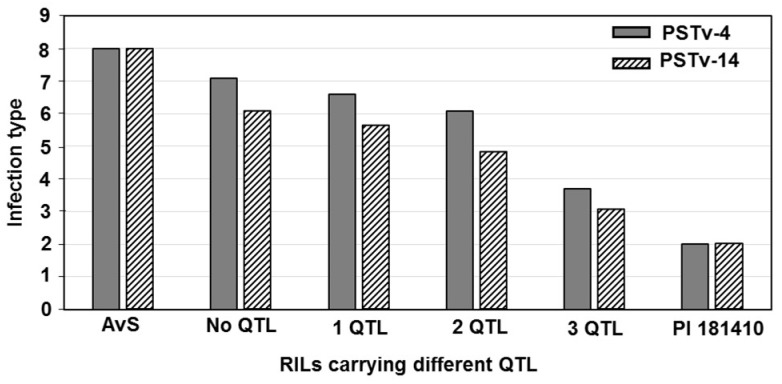
Comparison of mean infection type values of recombinant inbred lines (RILs) derived from cross AvS × PI 181410 containing different all-stage resistance QTL combination.

To estimate the effects of QTL combinations, the RILs were classified into different groups containing different QTL combinations based on the genotypes spanning the QTL intervals. According to the ASR QTL identified in the greenhouse seedling tests, RILs were classified into four groups (Figure 5). Although these QTL contributed minor effects to reduce the stripe rust to specific races including PSTv-4 and PSTv-14 in the greenhouse conditions, the RILs containing more QTL showed higher resistance than those containing few QTL.

**Figure 6:**
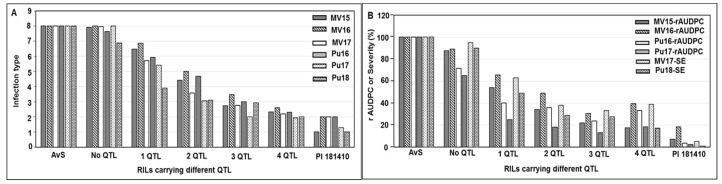
Comparison of mean infection type (**A**) and rAUDPC/severity (SE) (**B**) values of recombinant inbred lines (RILs) derived from cross AvS × PI 181410 containing different HTAP resistance QTL combination.

The similar trends were also found for HTAP resistance. Four QTL for HTAP resistance were identified in PI 181410. Based on the markers for these QTL, RILs were classified into five groups (Figure 6). *QYrPI181410.wgp-4BL* had a higher effect than *QYrPI181410.wgp-1BL*, *QYrPI181410.wgp-5AS*, and *QYrPI181410.wgp-5BL.2*. The resistance level was greatly increased when the major QTL *QYrPI181410.wgp-4BL* was in combination with minor QTL *QYrPI181410.wgp-1BL* and/or *QYrPI181410.wgp-5BL.2*. The RILs containing one of the QTL showed a lower IT and rAUDPC than those without any QTL. The RILs containing more resistance QTL had a higher level of resistance (lower IT and/or rAUDPC values) than those containing a smaller number of QTL (Figure 6A,B). These results indicated that the combinations of different QTL increased the level of resistance through additive interactions.

### 2.7. Kompetitive Allele Specific PCR (KASP) Markers for the Major-Effect QTL

As *QYrPI181410.wgp-4BL* was the major-effect QTL for HTAP resistance identified in PI 181410 and detected in all greenhouse and field tests, KASP markers were developed for this QTL. Five KASP markers were successfully converted from their original SNP markers (Table 6). Three of five KASP markers, *KIWB81016*, *KIWB6961*, and *KIWB80,* which were developed from SNP markers *IWB81016*, *IWB6961*, and *IWB80*, respectively, were confirmed to be polymorphic between PI 181410 and AvS. When tested on the RIL population, the KASP markers produced the same genotypes as their original SNP markers among the individual RILs.

The three polymorphic KASP markers, were used to determine their polymorphisms in 155 wheat cultivars and breeding lines from the US (Table S3). These markers had polymorphism rates 45.8%, 69.0%, and 100%, respectively. As expected, none of the 155 wheat accessions had the same marker haplotype of PI 181410 for the three KASP markers. This result indicated that *QYrPI181410.wgp-4BL* was absent in the collection of US wheat cultivars and breeding lines, and the KASP markers in combination, or individual marker KIWB80, should be useful in MAS for incorporating this QTL into new wheat cultivars.

## 3. Discussion

Wheat landrace PI 181410 has been consistently showing high-level resistance to stripe rust in the US Pacific Northwest region since 2006. The four-way tests conducted with predominant and most virulent races of *Pst* in both seedling and adult-plant stages at both LT and HT profiles showed that PI 181410 has both race-specific ASR and high-level HTAP resistance. Using the high-density genetic maps and multiple environmental tests for QTL mapping, we identified three QTL in PI 181410 for ASR, *QYrPI181410.wgp-4AS*, *QYrPI181410.wgp-4BL*, and *QYrPI181410.wgp-5BL.1*. In addition to the QTL on 4AS, we identified three more QTL, designated as *QYrPI181410.wgp-1BL*, *QYrPI181410.wgp-5AS*, and *QYrPI181410.wgp-5BL.2* in PI 181410 conferring resistance observed at the adult-plant stage in the fields. Interestingly, we also detected two QTL, designated as *QYrAvS.wgp-2BS* and *QYrAvS.wgp-2BL*, from AvS with minor effects only on IT at Mount Vernon and Pullman, respectively. Each of the QTL were located within a relatively small genomic region.

*QYrPI181410.wgp-1BL* associated with minor HTAP resistance was detected at the distal end of the long arm of 1B. *Yr29* is also located at the distal end of 1BL [13]. At the *Yr29* location, eleven QTL were identified with different phenotypic effects [14]. Six of the eleven QTL have been reported to provide significant effects on stripe rust resistance in multiple environmental tests, such as *QYr.sun-1B*, *QYr.jic-1B*, *QYr.tam-1B*, *QYr.cim-1BL*, *QYr-1B*, *QYr.cim-1BL*, and *QYr.1BL* [15,16,17,18,19,20]. Other QTL including *QYrPst.jic-1B* were reported to have minor effects on stripe rust resistance [14,21]. Based on the chromosome regions, *QYrPI181410.wgp-1BL* is likely *Yr29*. The present study and previous studies suggests that the long arm of chromosome 1B carries multiple genes for resistance to stripe rust.

Two minor-effect QTL, *QYrAvS.wgp-2BS* and *QYrAvS.wgp-2BL*, were identified from susceptible parent AvS. *QYrPI181410.wgp-2BS* was mapped close to the distal end of the short arm. According to previous studies, two QTL including *QYr.inra-2BS* [22] and *QYr.wgp-2B.1* [23] were mapped at the same region as *QYrAvS.wgp-2BS*. *QYr.inra-2BS* contributed moderate resistance to stripe rust at both seedling and adult-plant stages [22]. Identified in several US Pacific Northwest wheat cultivars by genome-wide association mapping, *QYr.wgp-2B.1* contributed adult-plant resistance to stripe rust and was significantly associated with both IT and SE in several environments including Pullman and Mount Vernon, Washington [23]. However, *QYrPI181410.wgp-2BS* was only associated with IT in Mount Vernon. Thus, *QYrAvS.wgp-2BS* is likely a locus different from *QYr.inra-2BS* and *QYr.wgp-2B.1*. *QYrAvS.wgp-2BL* was only detected using one set of IT data in a single field condition in Pullman. It was located at the distal end of 2BL. Rosewarne et al. [17] reported a minor QTL affecting adult-plant resistance in AvS flanked by simple sequence repeat (SSR) markers *Xgwm1027* and *Xgwm619* at the same location on chromosome 2BL as *QYrAvS.wgp-2BL*. Since their QTL and *QYrAvS.wgp-2BL* were detected in the same wheat genotype in the same chromosomal location, they should be the same gene.

*QYrPI181410.wgp-5AS* had a minor effect on reducing disease severity in PI 181410 and was located at the telomere of chromosome 5AS. As no stripe rust resistance QTL have been reported at this location, the *5AS* QTL is a new QTL for resistance to stripe rust.

Two QTL, *QYrPI181410.wgp-5BL.1* and *QYrPI181410.wgp-5BL.2*, from PI 181410 were mapped to different locations on chromosome 5BL. These QTL conferred resistance to stripe rust at different plant growth stages. *QYrPI181410.wgp-5BL.1*, associated with ASR, was located at the middle region of 5BL, while *QYrPI181410.wgp-5BL.2*, associated with HTAP resistance, was mapped to the distal end of 5BL. Five previously reported QTL, including *QYr.caas-5BL.1* from Libellula [24], *QYrdr.wgp-5BL.2* [25], *QYrco.wpg-5B* [26], *QYr.jirc-5BL* [27], and *QYr.inra-5BL.1*, were at the chromosomal regions similar to *QYrPI181410.wgp-5BL.1* based on their flanking markers. All these QTL exhibit adult-plant resistance to multiple races, however, *QYrPI181410.wgp-5BL.1* conferred ASR to races PSTv-4 and PSTv-14. Based on the different types of resistance, *QYrPI181410.wgp-5BL.1* may be different from those known QTL, but further work is needed to determine their genetic relationships. Two QTL, *QYr.caas-5BL.2* flanked by *Xbarc142* and *Xgwm604* [24] and *QYr.caas- 5BL.3* flanked by SSR markers *Xwmc75* and *Xbarc275* [28], were mapped to the distal end of 5BL [14]. Compared with the consensus map [29], *QYrPI181410.wgp-5BL.2* should be close to the far distal end of the 5BL and not in the same region as the above two QTL. Thus, *QYrPI181410.wgp-5BL.2* is likely a new QTL for stripe rust resistance.

Among the QTL identified in PI 181410, *QYrPI181410.wgp-4BL* was the most effective. It was consistently detected using all 16 sets of phenotypic data collected from all field tests and both seedling and adult-plant tests in the greenhouse. Its effect was much stronger in the adult-plant stage in the fields and at HT in the greenhouse test than the seedling stage at LT, and therefore, it should be considered as a HTAP resistance gene. *QYrPI181410.wgp-4BL* was mapped to an interval of 9.0 cM (67.0~76.0 cM) close to the centromeric region of 4BL according to the consensus map [29,30]. The overlap of the genomic regions detected by all 16 data sets narrowed the QTL to 4.0 cM between 68.0 and 72.0 cM positions (Table 5, Figure 4). In previous studies, *Yr50* and *Yr62* were also mapped to the same location on 4BL as *QYrPI181410.wgp-4BL*. The dominant resistance gene, *Yr50*, derived from *Thinopyrum intermedium*, was mapped on 4BL and flanked by SSR markers *Xbarc1096* and *Xwmc47*, and *Yr50* exhibited ASR to all tested Chinese *Pst* races and eight US predominant *Pst* races including PSTv-40 [31,32]. In contrast, *QYrPI181410.wgp-4BL* is from common wheat landrace PI 181410 and confers strong HTAP resistance with only a minor effect at seedling stage. The differences clearly indicate that *QYrPI181410.wgp-4BL* is different from *Yr50*. *Yr62*, from Portuguese spring wheat variety PI 192252, was flanked by SSR markers *Xgwm192* and *Xgwm251* on 4BL [32]. This gene provides a higher level HTAP resistance (40%–60% of phenotypic variation) than *QYrPI181410.wgp-4BL* (29.2%–46.9%). *Yr62* is not effective at the seedling stage. Thus, *QYrPI181410.wgp-4BL* is likely different from *Yr62*. Further studies are needed to determine their relationship using the allelism test approach.

Two major QTL, *QPst.jic-4B* and *QYrva.vt-4BL*, were also mapped around the centromeric region on 4BL [21,23]. *QPst.jic-4B*, identified from a German winter wheat cultivar Alcedo, was flanked by amplified fragment length polymorphism (AFLP) marker *S24M37_130* and SSR marker *Xcfd39* and peaked at *Xwmc692* on 4BL [21]. *QYrva.vt-4BL*, identified from a soft red winter wheat VA00W-38, was flanked by SSR markers *Xwmc692* and *Xgwm149* on 4BL [33]. Both *QPst.jic-4B* and *QYrva.vt-4BL* explained less effect (17.9%–28.9% and 19.3%, respectively) of the phenotypic variation. Thus, *QYrPI181410.wgp-4BL* is unlikely the same as any of these two QTL in winter wheat cultivars. As *QYrPI181410.wgp-4BL* is the most useful among the QTL detected in the present study, we developed three KASP markers for this QTL. The KASP markers performed in the same way as the original SNP markers between the parents and among the RILs, and they were highly polymorphic when tested with 155 wheat cultivars and breeding lines from the US Pacific Northwest. None of the wheat materials have the *KIWB80* allele or the PI 181410 haplotype of the three markers. Therefore, the KASP marker *KIWB80* or the combination of the three markers can be used in MAS for incorporating the effective QTL into various wheat backgrounds for developing new cultivars with durable, high-level resistance to stripe rust.

## 4. Materials and Methods

### 4.1. Plant Materials

PI 181410, an Afghanistan spring wheat landrace with resistance to stripe rust, was used as the male parent in crossing with Avocet S (AvS), a stripe rust susceptible Australian spring wheat genotype, as a female parent. A recombinant inbred line (RIL) population of the cross was developed using the single-seed decent method. A total of 170 RILs of the F_5_, F_6_, F_7_, and F_8_ generations were used for phenotyping the response to stripe rust in different years and locations, and the first 114 F_7_ RILs of the F_7_ generation were used for SNP genotyping. A collection of 155 US Pacific Northwest spring and winter wheat cultivars and breeding lines was used to determine polymorphisms of the KASP markers developed in this study.

### 4.2. Evaluation of PI 181410 and RILs for Stripe Rust Reaction in the Greenhouse

To characterize the stripe rust resistance in PI 181410, seedlings and adult plants were tested with five US predominant or most virulent races of *Pst*, PSTv-4 (isolate 2013–445), PSTv-14 (isolate 2012–116), PSTv-37 (isolate 2012–114), PSTv-40 (isolate 2009–78), and PSTv-51 (isolate 2011–366) [9,10], following the procedures as previously described [7,34]. Briefly, about 10–15 seeds of each line were planted in one pot in a plastic tray (53.43 × 27.5 × 6.10 cm, containing 72 pots) filled with soil mixture. Seedlings at two-leaf stage [growth stage (GS) 10] [35] were inoculated with urediniospores of a selected race mixed with talc at the ratio of 1:20. The inoculated plants were incubated in a dew chamber at 10 °C in darkness for 24 h for urediniospores to germinate and infect the plants, and then transferred to a growth chamber at a diurnal cycle of low-temperature (LT) gradually changing from 4 °C at 2:00 a.m. to 20 °C at 2:00 p.m. with a 16 h light/8 h dark photoperiod. For high-temperature (HT) tests, after incubating in the dew chamber for 24 h at 10 °C in darkness, the inoculated plants were placed in a growth chamber at a diurnal temperature cycle gradually changing from 10 °C at 2:00 a.m. to 30 °C at 2:00 p.m. with the same photoperiod cycle. For adult-plant tests at both LT and HT profiles, 3 plants grown in a pot (15 × 15 × 18 cm) to the boot stage (GS 45) were inoculated, incubated, and grown under the same conditions as described above. For each race–temperature test, a total of 9 plants in 3 pots were evaluated. Stripe rust infection type (IT) of each plant was scored at 18 to 21 days after inoculation using the 0 to 9 scales [36]. For the seedling tests, ITs of the second leaves were recorded while those of the flag leaves were recorded for the adult-plant tests. Seedlings or adult plants of AvS were included as the susceptible check in each stage–temperature test.

Races PSTv-4 and PSTv-14 of *Pst* were used for testing the 170 F_7_ RILs at the seedling stage at the LT profile (4–20 °C) with three biological replicates based on their relative avirulence on the seedlings of PI 181410. In these tests, PI 181410 and AvS were included as the resistant and susceptible checks, respectively, and the 18 *Yr* single-gene differentials [9] were included to ensure the correct race. PSTv-37 and PSTv-40 were used to test the adult plants of 170 RILs at the HT profile (10–30 °C) based on their virulence on seedlings at LT and avirulence on adult plants of PI 181410 at HT. PI 181410 and AvS were also included as the resistant and susceptible checks, respectively. The inoculation, incubation, growth of plants, and IT data record were the same as described above for the seedling LT and adult plant HT tests for the parental lines.

### 4.3. Evaluation for Stripe Rust Responses of PI 181410 and RILs in the Fields

The stripe rust responses of PI 181410, together with other wheat genotypes, were tested under natural *Pst* infections in field nurseries at Mount Vernon in northwestern Washington and Pullman in southeastern Washington since 2006. The two locations are about 500 km apart with different climate conditions and different *Pst* races [9,10]. The 170 F_5_, F_6_, F_7_, or F_8_ RILs were consecutively phenotyped in both locations in 2015, 2016, 2017, and 2018, respectively. The parental lines and RILs were planted in a randomized block design with three replications. About 20–30 seeds of each line were planted in a 50 cm row with 20 cm between rows. As a susceptible check and spreader, AvS was planted every 20 rows and around the field. Stripe rust IT and severity (SE) were recorded three times at the tillering stage (GS 30–31), booting stage (GS 45), and flowering to grain-filling stages (GS 60–70). Severity was recorded as the percentage of leaf area infected. The SE data were used to calculate the area under the disease progress curve (AUDPC) [37], which was then converted to relative AUDPC (rAUDPC) as percentage of the mean AUDPAC (as 100%) of susceptible parent AvS.

### 4.4. Statistical Analyses of the Phenotypic Data

Broad-sense heritability (*H*^2^) of phenotypic traits was calculated using the heritability package of R program following the formula *H*^2^ = *δ*^2^(G)/[*δ*^2^ (G)+ *δ*^2^ (G*L)/*l*+ *δ*^2^ (G*Y)/*y*+ *δ*^2^ (E)/*ly*], where *δ*^2^(G) = genotypic variance, *δ*^2^(G*L) = variance of genotype by location interaction, *δ*^2^(G*Y) = variance of genotype by year interaction, *δ*^2^(E) = residual variance, *l* = number of location, and *y* = number of years (https://cran.r-project.org/web/packages/heritability/heritability.pdf).

The analysis of variance (ANOVA) was performed for the IT data, SE, and rAUDPC using the *aov* function of the R program (version 3.5.2, https://www.r-project.org) to determine the effects of genetic and environmental factors and their interactions. Correlation coefficients were calculated using the correlation function in Excel for rAUDPC and IT data set to compare population responses across the four field environments and two greenhouse tests.

### 4.5. Genotyping the Parental Lines and RILs

The parental lines and F_7_ RILs were planted in the greenhouse for DNA extraction. Genomic DNA was extracted from the leaves harvested from a single plant of each F_7_ RIL using the CTAB (cetyl trimethylammonium bromide) method [38]. DNA quality was determined using 0.8% agarose gel electrophoresis, and DNA concentration was measured using a NanoDrop ND-1000 (Thermo Scientific, Wilmington, DE, USA). Each DNA sample was diluted to 50 ng/µL for use in genotyping. Out of the 170 F_7_ RILs, the first 114 RILs and parental lines were genotyped using the 90K Illumina^®^ iSelect wheat SNP assay [39] at the USDA-ARS Biosciences Research Laboratory, Fargo, ND. The reduced number of RILs genotyped was due to a budget limitation. SNP calling and clustering were determined using the functions of software GenomeStudio v2011.1 (Illumina, San Diego, CA, USA) based on the described genotypic data. Monomorphic SNPs, SNPs with more than 10% missing data, and RILs with 30% missing data were removed before further analyses.

### 4.6. Genetic Map Construction and QTL Mapping

SNP markers polymorphic between the parents were tested for the goodness of the fit to the 1:1 segregation ratio among the RILs using the chi-squared test in JoinMap 4.1 (https://www.kyazma.nl/index.php/JoinMap/Manual/). SNP markers that did not fit the 1:1 ratio (*p* < 0.01) were excluded for further analyses.

A genetic map was constructed using software JoinMap 4.1 [40] with the Kosambi mapping function [41]. The threshold value of logarithm of odds (LOD) score of 3.0 was applied to select the genetic linkage groups. The corresponding chromosome of each linkage group was determined based on the chromosome locations of SNPs from the 90K array [39]. The graphic genetic maps were generated using software MapChart v2.3 [42].

The IT data of both seedling stage and adult-plant stage in each year–location environment were used directly for QTL analysis. rAUDPC data were used for QTL mapping if the test had three sets of SE data. If only one set of SE data in the adult-plant stage was collected, as in the case of 2018 Pullman location, the SE data were also directly used for QTL mapping. QTL analysis was performed using both single-marker analysis (SMA) and composite interval mapping (CIM) with the Haley–Knott regression (HK) method in the R/qtl package of RStudio version 1.0.143 [43]. For QTL mapping, the probabilities of association between the genotypes and phenotypes were calculated using the function *calc.genoprob* with a step size of 1 cM distance and a genotype error of 0.01. SMA was performed using the *scanone* function and followed by CIM using the *cim* function. For each set of phenotypic data, a permutation test was performed using the *n.perm* function (*n* = 1000) to obtain a genome-wide LOD significance threshold. The resistance QTL interval with 95% confidence was obtained using the *bayesint* function. SNP markers closest to the QTL peak were identified using *find.marker*. The effect of each QTL was estimated using the *plotPXG* function. For the phenotypes with more than one QTL identified, the effect of each QTL was estimated using the multiple QTL mapping method in the R/qtl package [44]. The additive effect for each QTL was estimated using the *get.ests = TRUE* function. The interaction between two QTL was calculated using the function *addint* in the multiple QTL mapping method.

### 4.7. Developing KASP Markers

SNP markers flanking or at the peaks of the identified resistance QTL were selected for developing KASP markers using the information provided by PolyMarker (http://polymarker.tgac.ac.uk). Primers of KASP markers were synthesized by Sigma-Aldrich Inc. (Saint Louis, MO, USA). The KASP markers were validated using the parental lines PI 181410 and AvS and the RILs. The PCR reaction, amplification, and detection for KASP markers were conducted following the previously described method [45]. The PCR assay was performed in a LightCycler^®^ 480 System (Roche Applied Science, Indianapolis, IN, USA) using the endpoint genotyping analysis. To determine the polymorphisms of the KASP markers in various wheat backgrounds, 155 spring and winter wheat cultivars and breeding lines from the US Pacific Northwest were tested with the markers for the major effective QTL.

## 5. Conclusions

This study determined that the spring wheat landrace PI 181410, originally from Afghanistan, possesses both race-specific ASR and nonrace-specific HTAP resistance to stripe rust. Three QTL conferring ASR in PI 181410 were mapped to chromosomes 4BS, 4BL, and 5BL; and four QTL (including the 4BL QTL detected in the seedling stage) conferring HTAP resistance were mapped on chromosomes 1BL, 4BL, 5AS, and 5BL. The 1BL QTL was identified as *Yr29*, and the four other QTL in PI 181410 are potentially new. Two QTL were mapped on 2BS and 2BL from the susceptible parent AvS with minor effects on resistance in the field. The 2BL QTL is the same as a previously mapped QTL in the same variety, while the 2BS QTL is a potentially new locus. As the 4BL QTL was the most effective among the identified QTL and potentially new, three KASP markers (*KIWB81016*, *KIWB6961*, and *KIWB80*) were developed from its associated SNPs. These markers, especially *KIWB80*, were highly polymorphic among the US Pacific Northwest wheat cultivars and breeding lines. The resistant germplasm, identified QTL, and KASP markers for the most effective QTL are useful for developing wheat cultivars with durable, high-level resistance to stripe rust.

## Figures and Tables

**Table 1:** Stripe rust infection types of PI 181410 to five predominant or most virulent races of *Puccinia striiformis* f. sp. *tritici* in the United States tested in seedling and adult-plant stages at low (4–20 °C) and high (10–30 °C) temperature profiles.

Growth Stage	Temperatur Profile	Infection Type ^a^				
		PSTv-4	PSTv-14	PSTv-37	PSTv-40	PSTv-51
Seedling	Low	3	5	8	8	8
	High	2	2	7	2	2
Adult-plant	Low	2	2	2	2	2
	High	2	2	2	2	2

^a^ For comparison, AvS had infection type 8 or 9 in all of the tests.

**Table 2:** Analysis of variance (ANOVA) for relative area under the disease progress curve (rAUDPC) and infection type data for a recombinant inbred line (RIL) population derived from cross AvS × PI 181410 in different field environments of Pullman and Mount Vernon, WA in 2015, 2016, and/or 2017.

	rAUDPC				Infection Type			
Variance	*df*	MS	F	*p*	*df*	MS	F	*p*
RIL	162	4578	26.8	<0.0001	162	48.7	43.6	<0.0001
Environment	3	79480	465	<0.0001	3	254	227.2	<0.0001
RIL × Environment	485	671	3.93	<0.0001	484	6.1	5.5	<0.0001
Error	1268	171			1263	1.12		

**Table 3:** Correlation coefficients of infection type (IT) and relative area under the disease progress curve (rAUDPC) of the recombinant inbred lines of AvS × PI 181410 among the field tests in adult-plant stage in the fields under natural infections of *Puccinia striiformis* f. sp. *tritici*.

	Correlation Coefficient ^b^			
Test ^a^	MV15	MV16	Pu16	Pu17
MV15	0.80 ***	0.76 ***^c^	0.69 ***	0.67 ***
MV16	0.60 ***	0.86 ***	0.63 ***	0.57 ***
Pu16	0.67 ***	0.69 ***	0.53 **	0.61 ***
Pu17	0.67 ***	0.79 ***	0.68 ***	0.68 ***

^a^ MV = Mount Vernon and Pu = Pullman; and 15 = 2015, 16 = 2016, and 17 = 2017. ^b^ the left lower values are for comparing IT and the right upper values are for comparing rAUDPC values in different environments; and the diagonal four values are for comparing IT and rAUDPC in the same environments. ^c^ ** significant at *p* < 0.001 and *** significant at *p* < 0.0001.

**Table 4:** Quantitative trait loci (QTL) associated with stripe rust all-stage resistance detected in wheat genotype PI 181410 using the recombinant inbred line population derived from AvS × PI 181410.

QTL	Phenotypic Data Set ^a^	Closest Marker	Peak Position	LOD ^b^	QTL Interval (cM)	R^2^ (%)^c^	Additive Effect
***QYrPI181410.wgp-4AS***	PSTv-4-IT	*IWB9590*	43.0	3.3	41.0–45.0	11.2 ***	0.5
	PSTv-14-IT	*IWB9590*	43.0	2.7	40.0–44.0	5.4 **	0.4
***QYrPI181410.wgp-4BL***	PSTv-4-IT	*IWB6961*	71.0	3.6	68.0.0–88.0	8.6 **	0.5
	PSTv-14-IT	*IWB6961*	71.0	2.5	70.0–90.0	11.0 **	0.8
***QYrPI181410.wgp-5BL.1***	PSTv-4-IT	*IWB73753*	211.0	4.7	203.0–213.0	15.1 ***	0.6
	PSTv-14-IT	*IWB73753*	211.0	4.5	203.0–213.0	14.0 ***	0.7

^a^ In the phenotypic data sets, IT = infection type, the disease reactions of RILs were tested using two races, PSTv-4 and PSTv-14, in greenhouse condition. ^b^ LOD = logarithm of odds. ^c^ R^2^ = the percentage of phenotypic variation the QTL explained.

**Table 5:** Quantitative trait loci (QTL) associated with high temperature adult-plant resistance (HTAP) to stripe rust detected in wheat genotype PI 181410 and AvS using the recombinant inbred line population derived from AvS × PI 181410.

QTL	Phenotypic Data Set ^a^	Flanking Markers	Peak Marker	Peak Position	LOD ^b^	Interval of QTL (cM)	R^2^ (%) ^c^	Additive Effect ^d^
***QYrPI181410.wgp-1BL***	MV15-IT	*IWB6507, IWB72918*	*IWB6507*	160	6.4	159.0–165.0	10.5 ***	1.0
	MV16-IT	*IWB3715, IWB9772*	*IWB6507*	158	2.9	154.0–173.0	14.2 ***	1.0
	MV15-rAUDPC	*IWB6507, IWB73714*	*IWB6507*	160	3.6	159.0–168.0	7.3 ***	7.8
	MV17-IT	*IWB6507, IWB72918*	*IWB6507*	160	4.5	159.0–166.0	10.6 ***	0.9
	Pu16-IT	*IWB3715, IWB25646*	*IWB13472*	157	4.2	154.0–162.0	5.8 ***	0.5
***QYrAvS.wgp-2BS***	MV15-IT	*IWB13249, IWB11101*	*IWB11101*	18.0	3.1	12.0–20.0	5.6 ***	−0.4
	MV16-IT	*IWB13249, IWB11101*	*IWB11101*	18.0	3.0	12.0–20.0	5.0 ***	−0.5
***QYrAvS.wgp-2BL***	Pu16-IT	*IWB51607, IWB47487*	*IWB10669*	114.0	4.0	109.0–118.0	7.2 ***	−0.5
***QYrPI181410.wgp-4BL***	PSTv-37-IT	***IWB80^e^**, IWB40589*	*IWB70599*	70.1	10.8	68.0–73.0	43.7 ***	18.4
	PSTv-40-IT	***IWB80*** *, IWB72629*	***IWB691 ^e^***	70.6	11.7	67.0–72.0	46.9 ***	18.9
	MV15-IT	***IWB80*** *, IWB72629*	***IWB6961***	70.6	16.8	68.0–72.0	44.8 ***	1.8
	MV15-rAUDPC	***IWB80*** *, IWB72368*	*IWB70599*	71.0	17.2	68.0–74.1	45.4 ***	18.1
	MV16-IT	***IWB80*** *, IWB44800*	*IWB70599*	72.9	17.4	68.0–71.0	44.8 ***	1.9
	MV16-rAUDPC	***IWB80*** *, IWB51659*	*IWB44800*	71.7	15.1	68.0–73.0	50.0 ***	19.0
	MV17-IT	***IWB80*** *, IWB72629*	*IWB70599*	70.1	12.1	67.0–72.0	41.3 ***	1.7
	MV17-SE	***IWB80*** *, IWB72368*	*IWB36397*	71.9	9.7	68.0–74.0	43.7 ***	18.3
	Pu16-rAUDPC	***IWB80*** *, IWB44800*	*IWB70599*	70.1	12.2	68.0–71.0	44.0 ***	18.3
	Pu16-IT	***IWB80*** *, IWB44800*	***IWB6961***	70.5	14.4	68.0–71.0	29.2 ***	1.2
	Pu17-IT	***IWB80*** *, IWB81016 ^e^*	*IWB70599*	70.1	9.7	67.0–76.0	32.5 ***	1.7
	Pu17-rAUDPC	***IWB80*** *, IWB72368*	*IWB72368*	74.0	9.3	67.0–74.1	46.6 ***	18.4
	Pu18-IT	***IWB80*** *, IWB72629*	*IWB70599*	70.1	8.5	66.0–72.0	29.5 ***	1.8
	Pu18-SE	***IWB80*** *, IWB72368*	***IWB6961***	70.6	8.8	68.0–74.0	40.8 ***	17.7
***QYrPI181410.wgp-5AS***	MV17-SE	*IWB10594, IWB7246*	*IWB47184*	5.0	3.1	0–9.8	3.9 *	5.4
	Pu18-SE	*IWB10594, IWB7246*	*IWB47184*	2.7	3.4	0–8.0	4.2 *	5.6
***QYrPI181410.wgp-5BL.2***	MV15-IT	*IWB5343, IWB20628*	*IWB38584*	310.0	3.9	309.0–317.0	7.9 ***	0.6
	MV16-IT	*IWB59968, IWB20628*	*IWB76616*	311.0	6.7	306.0–317.0	4.8 *	0.6
	Pu16-IT	*IWB5343, IWB20628*	*IWB21151*	312.6	10.0	309.0–316.0	14.6 **	0.8
	Pu17-IT	*IWB59968, IWB20628*	*IWB38584*	310.8	4.8	307.0–317.0	17.3 ***	1.0
	Pu18-IT	*IWB59968, IWB7118*	*IWB20628*	317.0	5.9	307.0–321.0	14.0 ***	0.8

^a^ In the phenotypic data sets, MV = Mount Vernon and Pu = Pullman; 15 = 2015, 16 = 2016, and 17 = 2017; IT = infection type, rAUDPC = relative area under the disease progress curve, and SE = severity. ^b^ LOD = logarithm of odds. ^c^ R^2^ = the percentage of phenotypic variation the QTL explained. ^d^ additive effect: the negative value indicates that the resistance allele is from AvS (Avocet S). ^e^ The SNP markers highlighted in bold were successfully converted to KASP markers.

**Table 6:** Primer sequences of KASP markers for detecting the major stripe rust resistance QTL, *QYrPI181410.wgp-4BL*, from PI 181410 among 158 wheat cultivars or breeding lines.

SNP ID (90K)	KASP Primer	Sequences (5′−3′)
***IWB81016***	IWA7752A	GAAGGTGACCAAGTTCATGCTCCTATACAACAGAACACTGGGAACA
	IWA7752B	GAAGGTCGGAGTCAACGGATTCCTATACAACAGAACACTGGGAACG
	IWA7752C	AGAGTCGCCACGATCGTTTAACT
***IWB26339***	IWB26339A	GAAGGTGACCAAGTTCATGCTAGTTCTGGGTCGTCATTTTGT
	IWB26339B	GAAGGTCGGAGTCAACGGATTAGTTCTGGGTCGTCATTTTGC
	IWB26339C	ATGATTTGTGTTGTAGGGGGT
***IWB44800***	IWB44800A	GAAGGTGACCAAGTTCATGCTCCTCACAATGTTATGATTGACCAT
	IWB44800B	GAAGGTCGGAGTCAACGGATTCCTCACAATGTTATGATTGACCAC
	IWB44800C	CCTCGAAGCAACACGAACG
***IWB6961***	IWB6961A	GAAGGTGACCAAGTTCATGCTTGTTTGCTCTCATGAACGCTT
	IWB6961B	GAAGGTCGGAGTCAACGGATTTGTTTGCTCTCATGAACGCTC
	IWB6961C	CAGTTTCACATTCAACAGAAAGC
***IWB80***	IWB80A	GAAGGTGACCAAGTTCATGCTAAGAGGCCATGCTTCCCTA
	IWB80B	GAAGGTCGGAGTCAACGGATTAAGAGGCCATGCTTCCCTC
	IWB80C	AAGTTCAAGCATGGCAAGCA

## Supplementary Materials

Supplementary materials can be found at https://www.mdpi.com/1422-0067/21/2/478/s1.

## Abbreviations

*Pst*	*Puccinia striiformis* f. sp. *tritici*
ASR	All-stage resistance
HTAP	High-temperature adult-plant
SNP	Single nucleotide polymorphism
MAS	Marker-assisted selection
IT	Infection type
SE	Severity
LT	Low temperature
HT	High temperature
RIL	Recombinant inbred line
ANOVA	Analysis of variance
rAUDPC	relative area under the disease progress curve
QTL	Quantitative trait locus or loci
SSR	Simple sequence repeat
AFLP	Amplified fragment length polymorphism
GS	Growth stage
SMA	Single marker analysis
CIM	Composite interval mapping
LOD	Logarithm of odd
KASP	Kompetitive allele specific PCR
