# Supplementary material for: Genome-Wide Mapping of Quantitative Trait Loci Conferring All-Stage and High-Temperature Adult-Plant Resistance to Stripe Rust in Spring Wheat Landrace PI 181410
Source: Int J Mol Sci. 2020 Jan 12;21(2):478. doi: 10.3390/ijms21020478 (PMC7014124; doi:10.3390/ijms21020478)
Supplement: Supplementary file 1 [file ijms-21-00478-s001.zip › SupFigs.docx]

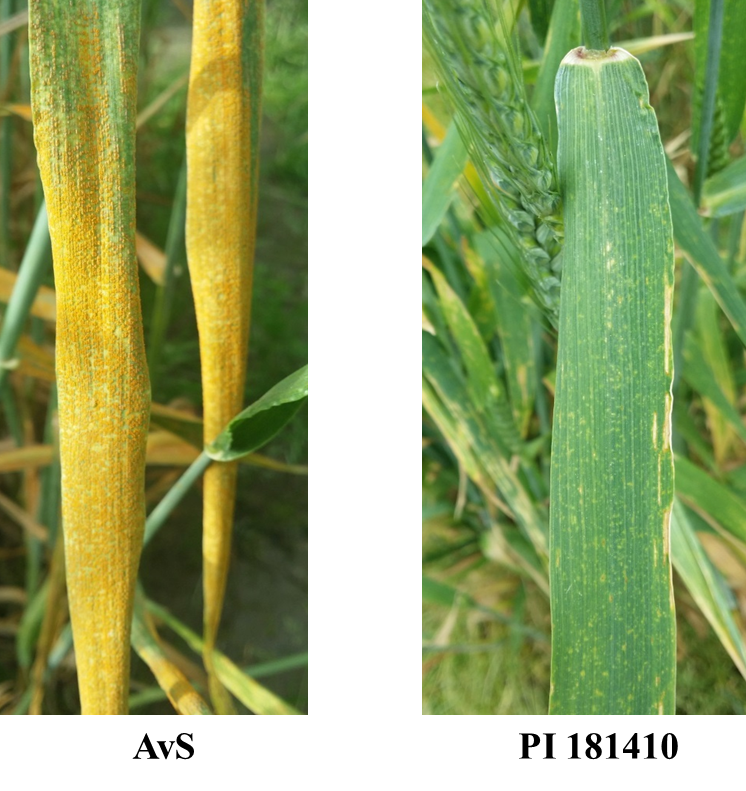


**Figure S1.** Photos of wheat genotypes AvS and PI 181410 showing susceptible and resistant reactions to stripe rust in the field of Pullman, Washington in 2017.


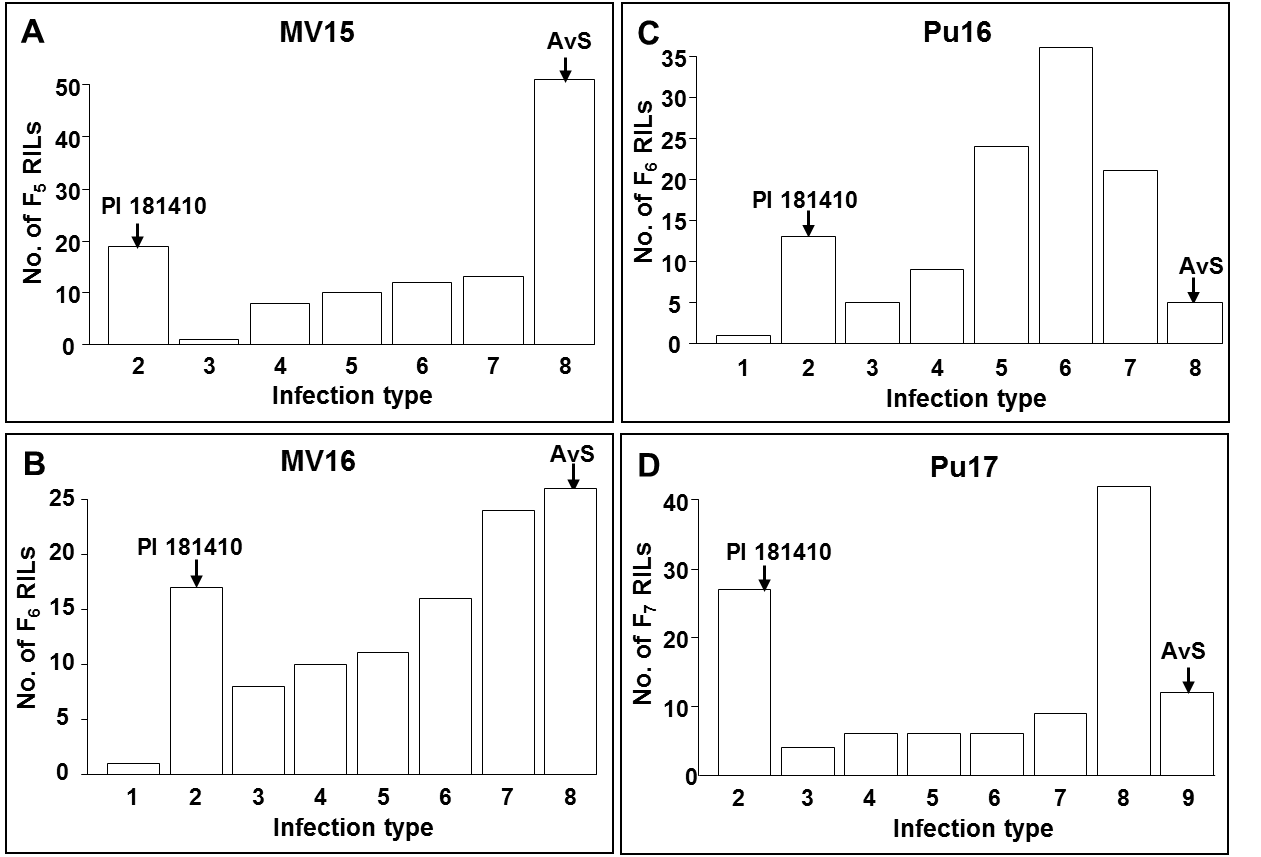


**Figure S2.** The distribution of stripe rust infection types among recombinant inbred lines (RILs) derived from the cross of AvS × PI 181410 tested in the fields of Mount Vernon (MV) in 2015 (15) and 2016 (16) and Pullman (Pu) in 2016 (16) and 2017 (17).


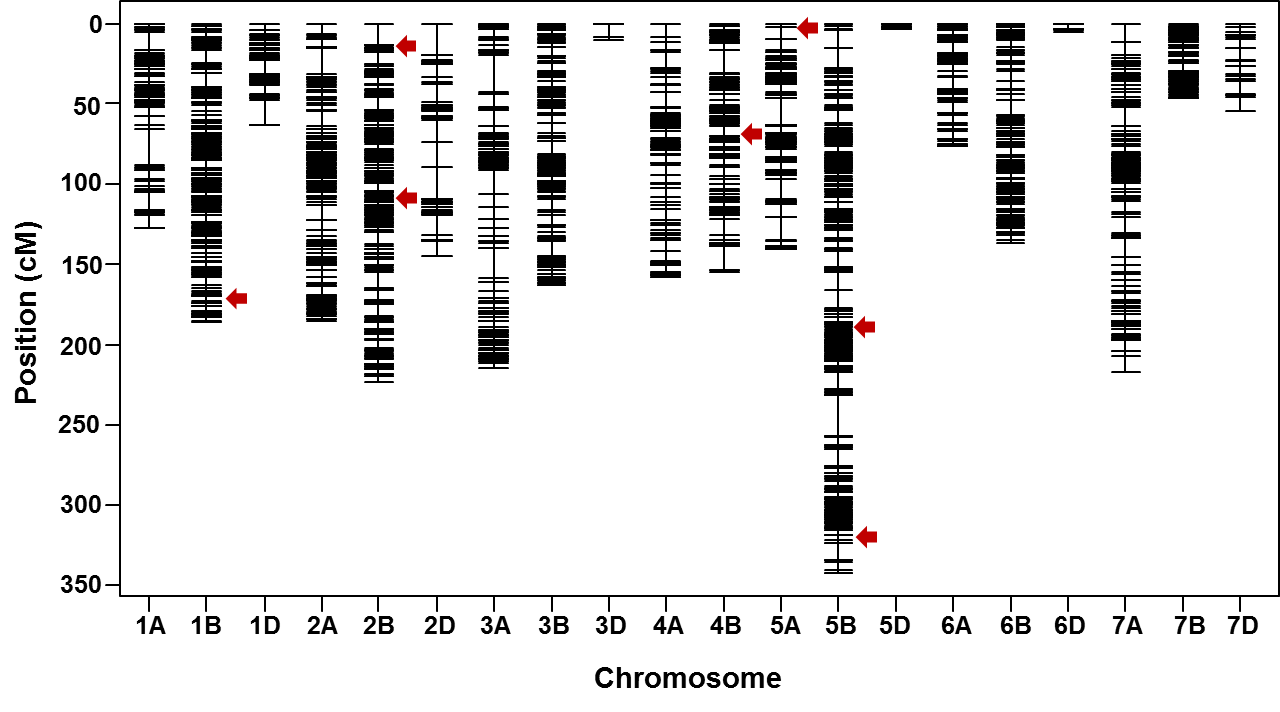


**Figure S3.** Genetic map consisting of linkage groups corresponding to 20 wheat chromosomes constructed using 114 recombinant inbred lines of cross AvS × PI 181410 genotyped with the 90K wheat SNP chip.
